# Mobile Genetic Elements as Central Drivers of Antimicrobial Resistance: Molecular Mechanisms, Evolutionary Ecology, One Health Implications and Control Strategies

**DOI:** 10.3390/antibiotics15040418

**Published:** 2026-04-20

**Authors:** Hemayet Hossain, Md. Hasan Ali, Tanvir Ahmad, Snigdha Sharmin Binte Sayeed, Md. Abdur Nur Sakib, Khadiza Akter Brishty, Md. Shah Jahan Saleh, Md. Mosharof Hosen, Shahabuddin Ahmed, Shihab Ahmed, Md. Shahidur Rahman Chowdhury, Md. Mahfujur Rahman

**Affiliations:** 1 Department of Veterinary Science and Animal Husbandry, Teesta University, Rangpur 5404, Bangladesh; hemayet.vabs@student.sau.ac.bd; 2 Faculty of Veterinary, Animal and Biomedical Sciences, Sylhet Agricultural University, Sylhet 3100, Bangladesh; hasan.dvm@student.sau.ac.bd (M.H.A.); ansakib.dvm@student.sau.ac.bd (M.A.N.S.); 3 Department of Medicine, Sylhet Agricultural University, Sylhet 3100, Bangladesh; tanvirahmad.vet@student.sau.ac.bd (T.A.); mosharof.vet@student.sau.ac.bd (M.M.H.); shahidur.vetmed@sau.ac.bd (M.S.R.C.); 4 Department of Microbiology and Hygiene, Bangladesh Agricultural University, Mymensingh 2202, Bangladesh; snigdhasharmin.bd16@gmail.com; 5 Department of Zoology (GSSC), University of Dhaka, Dhaka 1000, Bangladesh; brishtydu20@gmail.com; 6 Department of Livestock Production and Management, Sylhet Agricultural University, Sylhet 3100, Bangladesh; shahjahan.sau.dvm21@gmail.com; 7 Department of Animal Nutrition, Khulna Agricultural University, Khulna 9202, Bangladesh; drshahab@kau.edu.bd; 8 Antimicrobial Resistance Reference Laboratory (Research), Bangladesh Livestock Research Institute, Dhaka 1341, Bangladesh; shihab@blri.gov.bd

**Keywords:** mobile genetic elements, horizontal gene transfer, resistome dissemination, plasmids and integrons, One Health

## Abstract

Antimicrobial resistance (AMR) represents a global health crisis, driven largely by the mobility of resistance determinants through mobile genetic elements (MGEs). These include plasmids, integrons, insertion sequences, transposons, integrative and conjugative elements (ICEs), and prophages, which together facilitate horizontal gene transfer (HGT) across bacterial species and ecosystems. This review aims to provide a comprehensive synthesis of current knowledge on the types, mechanisms, ecological drivers, and impacts of MGEs in the dissemination of antibiotic resistance genes (ARGs). Methods involved critical evaluation of recent genomic, epidemiological, and ecological studies, alongside case studies of clinically significant resistance outbreaks. Findings highlight how MGEs function as hubs for ARG capture, recombination, and stabilization, enabling the emergence of multidrug-resistant (MDR) and extensively drug-resistant (XDR) pathogens. We also explored their interactions with ecological pressures such as antibiotics, heavy metals, and biocides, as well as their role in One Health transmission pathways. The significance of this study lies in linking molecular insights with applied strategies, including genomic surveillance, MGE-targeted inhibitors, phage therapy, and CRISPR-based interventions. Understanding MGEs is essential for designing effective interventions to mitigate AMR and protect global health.

## 1. Introduction

Antimicrobial resistance is now one of the greatest threats to global health. It makes modern medicine less effective and leads to greater sickness, death, and economic cost worldwide [1,2]. Without immediate action, approximately 10 million people will likely die annually by the year 2050 due to AMR and an estimated $100 trillion lost to the global economy [3].

AMR is a major clinical and economic issue worldwide. Economic damage ranges from $21,832 per case to over $3 trillion in lost GDP, while the extra cost of the healthcare system ranges from non-significant to $1 billion annually [4]. Enterobacteriaceae are resistant to third-generation cephalosporin; resistance is a prevalent feature in middle-income countries (MICs) [5]. AMR is responsible for almost 30% of neonatal sepsis deaths in sub-Saharan Africa, and therefore low-income countries (LICs) must bear the brunt [6].

Although certain bacterial lineages may develop intrinsic resistance due to spontaneous mutation, HGT facilitated by MGEs like plasmids, integrons, transposons, insertion sequences, ICEs, and prophages is the main cause of the spread of AMR worldwide [7,8]. Human mobility and international trade, environmental exposure to agricultural drainage and wastewater, and inadequate One Health integration permitting transmission across species are the main drivers of spread [9,10,11].

In addition to acquired mechanisms, almost exclusively by HGT and not mutation, AMR occurs by reason of intrinsic characteristics, i.e., membrane impermeability or the absence of drug targets [8,12]. MGEs like plasmids, transposons, integrons, and phages, which mobilize resistance genes across bacterial hosts [13], drive HGT through transformation (uptake of environmental DNA), transduction (phage-mediated gene transfer), and conjugation (plasmid-mediated cell-to-cell transfer) [14]; MGEs are therefore important agents of resistance transmission and possible targets for new antimicrobials.

Selfish DNA known as MGEs migrate both within and between genomes, transmitting genes of metabolism, stress resistance, virulence, and antibiotic resistance, thereby fueling microbial evolution [8,14]. ARGs are localized in MGE “hubs” such as integrons, ICEs, genomic islands, and plasmids, as reservoirs and facilitators of the transmission of cross-species resistance, as emerging from recent genomic surveillance (2020–2024) [12,13].

By mobilizing environmental reservoir genes into bacteria and driving fast adaptation under antibiotic-selective pressure, MGEs are the primary cause of AMR [8,15]. Through the integration of the microbiomes of human and animal hosts and the environment, they facilitate cross-species and cross-ecosystem transmission [16,17,18]. MGEs are networked as they fragment and modulate the resistome among ecosystems, following a “nested doll” modular organization [13,19,20].

There are significant knowledge gaps in MGEs, in particular, despite advancements in AMR studies. Developing MGEs, such as integrative and conjugative elements (ICEs), miniature inverted-repeat transposable elements (MITEs), phage-inducible chromosomal islands (PICIs), and CRISPR-associated transposons, increases genetic mobility and mediate ARG and virulence transfer, although their prevalence and processes are still poorly known [21]. In accordance with [22], phages have the capacity to disseminate a variety of ARGs through phage-plasmids; however, despite metagenomic breakthroughs, prevalence and usability remain inadequately understood.

Beyond Enterobacteriaceae, MGEs play equally critical roles in shaping antimicrobial resistance across diverse bacterial taxa. In non-Enterobacteriaceae, Proteobacteria such as *Pseudomonas aeruginosa* and *Vibrio cholerae*, integrons, genomic islands, and ICEs are key drivers of ARG acquisition and environmental persistence [8,9,11]. Similarly, Firmicutes, including *Staphylococcus aureus* and *Enterococcus faecium*, plasmids, transposons (e.g., Tn916/Tn1546), and prophages, facilitate the dissemination of resistance to glycopeptides and macrolides. *Actinobacteria* and environmental microbiomes further serve as reservoirs of ancient and emerging ARGs, highlighting the extensive taxonomic and ecological breadth of MGE-mediated resistance [13,16]. These taxa contribute significantly to the global resistome and serve as reservoirs for ARG emergence and interspecies transfer under the One Health framework [18].

Therefore, a comprehensive understanding of MGEs as central drivers of AMR is crucial for designing targeted control measures. Integrative approaches such as resistome mobilome mapping, metagenomics, and pangenomic analyses offer new opportunities to track gene flow and develop interventions such as MGE inhibitors, phage therapy, and CRISPR-based antimicrobials. This review aims to synthesize current knowledge on the diversity, mechanisms, ecological roles, and One Health implications of MGEs in antimicrobial resistance dissemination. By linking molecular insights with applied strategies, it seeks to highlight how understanding MGE biology can inform the next generation of AMR surveillance and mitigation strategies.

## 2. Types of Mobile Genetic Elements and Structural Features

Mobile genetic elements are pivotal drivers of HGT, enabling the acquisition and dissemination of ARGs among diverse bacterial populations. These elements vary in structure, host-range, and mechanisms of mobility, but collectively, they form an interconnected genetic network that accelerates resistance spread (Table 1).

**Figure 1:**
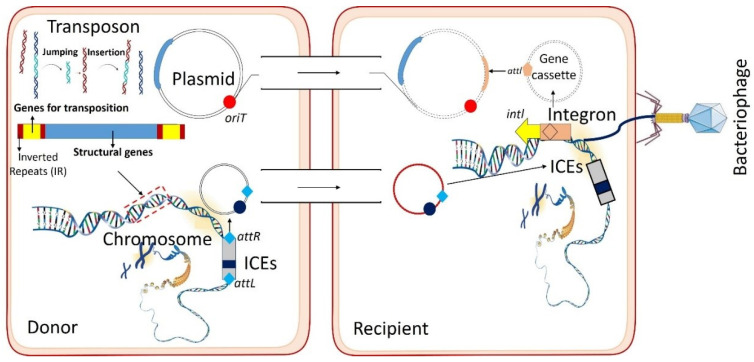
The function of mobile genetic elements (MGEs) in facilitating horizontal gene transfer (HGT) both within and between microbial cells. Bacteriophages insert their DNA into the host chromosome when they infect microbiological cells. Integrative and conjugative elements (ICEs) and plasmids are examples of conjugative elements that can be transferred across cells. ICEs can also mediate the mobilization of chromosomal genes and facilitate the recombination of transferred DNA within recipient genomes. Transposons (also known as insertion sequences [ISs]), integrons, and extra MGEs may be carried by these components. Figure 1 was generated using BioRender (http://www.biorender.com/).

Plasmids are self-replicating, circular double-stranded DNA molecules that often carry replication (*rep*), transfer (*tra*/*mob*), and stability genes [23] (Figure 1). These are ubiquitous across the bacterial domain and have been identified in a wide range of taxa, including both Gram-negative and Gram-positive bacteria, where they play central roles in horizontal gene transfer and adaptation [24]. Figure 1 illustrates the interconnected roles of MGEs in facilitating horizontal gene transfer across bacterial populations, highlighting the nested organization of integrons, transposons, and plasmids. Insertion sequences (ISs), typically <2.5 kb and encoding a transposase flanked by inverted repeats, facilitate ARG mobilization, for instance, *mcr-1* flanked by ISApl1 [8]. Similarly, transposons such as Tn4401 and Tn21 promote cut-and-paste or replicative transfer of *bla*_KPC_, *bla*_TEM_, and *erm* genes [3].

Integrons play a unique role by capturing, integrating, and expressing gene cassettes encoding aminoglycoside-modifying enzymes, *aadA*, or *dfr*, thereby acting as genetic platforms for ARG assembly [8]. Integrons, particularly class 1 and class 2, are known to carry over 130 different resistance gene cassettes [25], most of which are associated with resistance to the following:

- Aminoglycosides: *aadA* (aadA1, aadA2, aadA5), *aac* (aacA4, aac(6′)-Ib, aacC).
- Beta-lactams (ESBL): *bla*_IMP_, *bla*_OXA_ (*bla*_OXA-1_, *bla*_OXA-2_, *bla*_OXA-10_), *bla*_VIM_, *bla*_GES_.
- Trimethoprim: *dfrA* (dfrA1, dfrA5, dfrA7, dfrA12, dfrA17).
- Sulfonamides: *sul1*, *sul2*, *sul3*.
- Chloramphenicol: *catB*, *cmlA*.
- Macrolides/Lincosamides: *ereA*, *ereB*, *ermB*.
- Quaternary Ammonium Compounds (Disinfectants): *qacEΔ1*.
- Rifamycin: *arr*.

Integrative and conjugative elements (ICEs) and integrative mobilizable elements (IMEs) are chromosomal elements capable of excision and conjugative or plasmid-assisted transfer, often carrying multidrug resistance clusters in vibrios, streptococci, and enterococci [26,27]. Genomic resistance islands, such as AbaR in *Acinetobacter*, ensure long-term stability of ARGs within bacterial chromosomes [28]. Other MGEs, including prophages, ISCR elements, miniature inverted-repeat transposable elements (MITEs), and phage-inducible chromosomal islands (PICIs) also contribute to ARG movement, though often in specialized contexts [29]. Emerging systems such as super-integrons in vibrios and CRISPR-associated transposons highlight the evolutionary diversity of MGEs and their expanding roles in precise ARG mobilization [30,31].

**Table 1:** Mobile genetic elements (MGEs) involved in acquisition and dissemination of antibiotic resistance genes (ARGs).

MGE Type	Genetic Structural Features	Bacterial Host-Range	Role in Gene Transfer	Resistance Genes Commonly Associated	Representative Examples	References
Plasmids	Circular dsDNA; replication (*rep*), transfer (*tra*/*mob*), stability genes	Broad across Gram-negative and Gram-positive bacteria	Conjugation, mobilization, accessory gene carriage	*bla*_CTX-M_, *bla*_NDM_, *mcr*, *qnr*, *tet*, *sul*	IncF, IncI, IncX, IncA/C	[23,24]
Insertion Sequences (IS)	Short (<2.5 kb); transposase gene flanked by inverted repeats	Ubiquitous	Promote insertion of ARGs into plasmids/chromosomes	*bla*_NDM_ mobilization by IS26, *mcr-1* flanking by ISApl1	IS26, ISAba1, ISCR1	[8]
Transposons	Composite (IS-flanked) or Tn3-like; *tnpA*/*tnpR*	Gram-negatives, Gram-positives	Cut-and-paste or replicative transposition	*bla*_KPC_ (Tn4401), *bla*_TEM_ (Tn3 family), *erm* genes	Tn4401, Tn3, Tn21	[3]
Integrons	*intI* (integrase), *attI* recombination site, Pc promoter, gene cassette array	Enterobacteriaceae, *Pseudomonas*, *Acinetobacter*	Capture, integrate, and express gene cassettes	Aminoglycoside-modifying enzymes, *dfr*, *aadA*, *qnr*	Class 1 integrons	[8]
ICEs (Integrative and Conjugative Elements)	Chromosomally integrated; excision genes (*xis*/*int*), conjugation (*tra*), replication	Vibrios, Enterobacteriaceae, *Streptococcus*, *Enterococcus*	Conjugative transfer between distantly related bacteria	MDR clusters (*tet*, *sul*, *bla*, *floR*)	SXT/R391 ICEs	[26]
IMEs (Integrative and Mobilizable Elements)	ICE-like but lack full conjugation machinery; mobilization via relaxase (*mob*)	Gram-positive cocci (*Streptococcus*, *Enterococcus*)	Mobilized by conjugative plasmids/ICEs	Macrolide and tetracycline resistance	IME-Spn1	[26,32]
Genomic Islands (Resistance Islands)	Large chromosomal DNA blocks; often contain integrons, transposons, ARG clusters	MDR Gram-negatives (*K. pneumoniae*, *A. baumannii*, *E. coli*)	Long-term maintenance and clonal expansion	*bla*_NDM_, *bla*_OXA_, *armA*, *sul*, *tet*	AbaR (Acinetobacter), SGI1 (Salmonella)	[27,33]
Prophages	Bacteriophage genomes integrated in bacterial chromosome	Wide	Transduction of ARGs	*bla*_CTX-M_, *erm*, *mef*	*E. coli* ST131 phage-borne ARGs	[34,35]
ISCR Elements	Related to IS91 family; rolling-circle transposase (*orf513*)	Enterobacteriaceae	One-ended transposition mobilizing adjacent ARGs	*bla*_NDM_, *bla*_CTX-M_, *qnr*, *mcr*	ISCR1, ISCR2	[36]
MITEs (Miniature Inverted-repeat Transposable Elements)	Short (100–600 bp), inverted repeats, non-coding	Associated with Gram-negatives	Depend on other transposases; sometimes capture regulatory regions	Regulatory influence on ARG expression	Associated with *sul*, *tet*	[37,38]
PICIs (Phage-Inducible Chromosomal Islands)	Chromosomal islands hijacking helper phage machinery	*Staphylococcus aureus*, Streptococci	Phage-dependent mobilization	Virulence genes, some ARGs (*erm*)	SaPI1 (*S. aureus*)	[39]
Super-Integrons	Chromosomal arrays with hundreds of gene cassettes; large *intI* variants	*Vibrio*, aquatic bacteria	Reservoirs of novel ARGs under selection	Aminoglycoside, sulfonamide, trimethoprim resistance	Vibrio cholerae super-integron	[40]
CRISPR-associated Transposons	Transposons using CRISPR-Cas for guided integration (e.g., *Tn7-like* + CRISPR)	Emerging in Gram-negatives	Precise targeted mobilization of ARGs	Potentially any ARG linked to CRISPR-target	Tn7-CRISPR systems	[30,31]

## 3. Molecular Mechanisms of Acquisition and Mobilization

**Figure 2:**
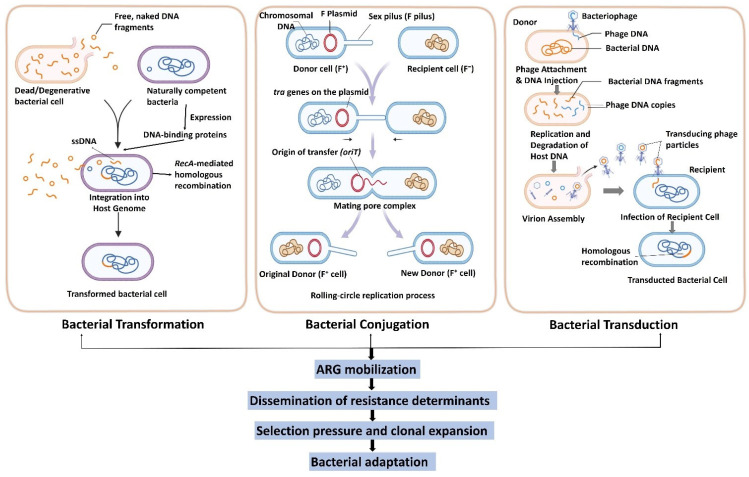
Mechanisms of mobile genetic elements in acquisition and mobilization of ARGs. Transformation: uptake of free DNA from the environment; integrated into host genome. Conjugation: direct DNA transfer via cell-to-cell contact; involves plasmids or chromosomal DNA. Transduction: DNA transfer by bacteriophages; viral packaging and infection lead to gene exchange. Consequences: spread of antibiotic resistance; genetic diversity and adaptation; clonal expansion under selection pressure. Figure 2 was generated using BioRender (http://www.biorender.com/).

Integrons, transposons, bacteriophages, and plasmids are among some MGEs that enable HGTs and the spread of ARGs among bacteria [8]. HGT depends on three mechanisms: conjugation, the transference of DNA by plasmids or ICEs from one cell to another (Figure 2) [41]; transformation, in which bacteria absorb DNA from their surroundings; and transduction, in which bacteriophages transfer bacterial DNA, e.g., ARGs, by a process referred to as transduction without direct contact [42]. This plasticity enhances bacterial adaptation to antibiotic pressure, highlighting the significant role MGEs play in developing and spreading resistance [8,43].

### 3.1. Conjugation: The Major Route of ARG Spread

Conjugation is an essential horizontal transfer mechanism of ARGs between bacterial species. It involves relaxase (Mob) proteins, which nick the transfer origin (*oriT*), *tra*/*trb* genes that mediate the formation of mating pairs, and the Type IV secretion system (T4SS) for DNA transmission. It transfers plasmids or chromosomal ICEs with ARGs and donor–recipient contact triggers this, usually through a pilus. The mobility of genes like *bla*_NDM_, *mcr-1*, and *qnr* via broad host-range plasmids like *IncP* and *IncA*/*C* confers resistance to carbapenems, colistin, and quinolones, which promotes the spread of ARG and leads to severe public health issues (Table 2) [8,13]. Representative conjugative systems are selected to illustrate major clinically relevant plasmid incompatibility groups rather than provide an exhaustive list.

### 3.2. Transformation: Uptake of Free DNA

Systems such as the com operon in *Streptococcus* and *Haemophilus* for DNA binding, absorption, and homologous recombination facilitate transformation, a natural HGT mechanism in which competent bacteria absorb ambient DNA [55,56]. It causes antimicrobial resistance such as *Acinetobacter baumannii* acquisition of *bla*_OXA_*,* carbapenemase genes and *Streptococcus pneumoniae* incorporation of modified PBPs [32,57]. Environment like soil, water, and biofilms support transformation, as ARG reservoirs can transfer to pathogens under selective pressure [58].

### 3.3. Transduction: Phage-Mediated Gene Transfer

Among the phage-mediated processes of HGT critical to the spread of antibiotic resistance is transduction. As [59,60] explains, it involves specialized transduction (specific loci), generalized transduction (random host DNA), and lateral transduction, a very effective ARG transfer system in *Staphylococcus aureus*. Prophages induce bacterial adaptability to antibiotic pressure by mobilizing genes such as *erm*, *mef*, and *bla*_CTX-M_ [61]. Transduction, once thought to be insignificant, is currently known to be a primary mode of ARG transmission among microbial community, according to metagenomic surveys [62].

### 3.4. Recombination Events in ARG Mobilization

Recombination of ARG mobilization is performed in a variety of ways. Gene cassettes are inserted into integrons by site-specific recombination using integrases such as *IntI* [8,63]. Transposase or recombinase-catalyzed reactions produce composite transposons that transfer ARGs from plasmids to chromosomes and vice versa, whereas during transformation homologous recombination incorporates DNA [8]. IS26 mediates the reconfiguration of multidrug resistance plasmids, thereby enhancing genomic plasticity and promoting the persistence of antibiotic resistance genes (ARGs), while the *bla*_KPC_-containing island within Tn4401 facilitates the dissemination of carbapenem resistance [64].

### 3.5. Genetic Rearrangements and Mosaic/Hybrid MGEs

MGE recombination produces mosaic structures that group genes related to motility, virulence, and resistance, increasing the adaptability of bacteria [8,65]. ICE–plasmid fusions create “super-MGEs” with wider host-ranges and hybrid plasmids including transposons, integrons, and prophages (Table 3) [26,66]. According to Moghadam et al. [67], these mosaics accelerate the spread of multidrug resistance in healthcare environments by triggering XDR plasmids in *Acinetobacter baumannii* and *Klebsiella pneumoniae*.

### 3.6. Molecular Drivers of Mobilization

Multiple factors drive ARG mobilization: recombinases and integrases assemble integrons and transposons, clustering resistance genes [8]; insertion sequences like *IS26* and *ISApl1* move genes like *bla*_NDM_ and *mcr-1* via plasmids and transposons [64,75]. ARG expression is induced by regulatory factors such as the Pc promoter and IS-derivative promoters; SOS response is triggered by stress or exposure to antibiotics, augmenting integrase and transposase activity [8,76,77]. These mechanisms are synergistic to promote resistance dissemination and genetic plasticity.

### 3.7. Co-Occurrence and Interactions of MGEs

MGEs that interact with each other result in ARG mobilization, which frequently occurs according to “nested doll” and mobilome network models in which the smaller ones nest within larger ones to maximize transfer [78]. Examples include transposons in ICEs that excise and conjugate [79], prophages transporting ARGs in genomic islands [80] and IS26 reshuffling integrons within plasmids [81]. According to genomic research, the majority of plasmid-borne ARG transfers are driven by MGE interactions, specifically IS26 [82]. To fight antibiotic resistance, one has to comprehend these interactions [83].

## 4. Roles of MGEs in Dissemination of Resistome

**Figure 3:**
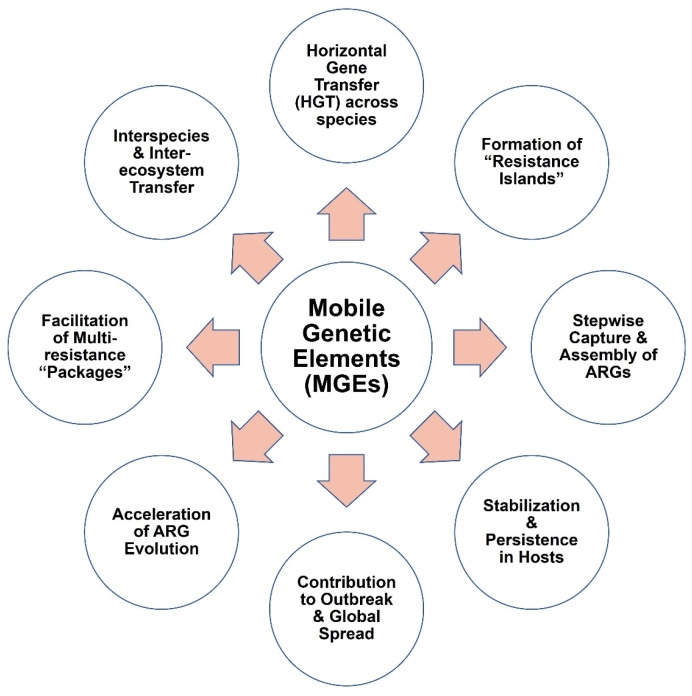
Role of mobile genetic elements (MGEs).

MGEs are responsible for the horizontal gene transfer of ARGs (Figure 3). Plasmids spread multidrug resistance, integrons acquire gene cassettes, and transposons/ISs move ARGs from one replicon to another [8,84,85]. ICEs connect conjugation and chromosomal integration. MGEs disseminate ESBL and carbapenemase genes to genera such as *Acinetobacter* and *Klebsiella* [86], and microbiomes are reservoirs of mobilized ARGs into infection, revealed by metagenomics [13,87]. Because MGEs accelerate the development of resistance, they remain significant targets for AMR control.

In the majority of ecological and clinical settings, MGEs have a significant role in acquiring, transferring, and disseminating ARGs (Figure 3). MGEs can carry out horizontal gene transfer between species, which is one of the most significant roles of MGEs that facilitates ARG diffusion occurring beyond clonal expansion. *Acinetobacter baumannii* and *Escherichia coli* can be transfected by plasmid-borne *bla*_NDM-1_ from *Klebsiella pneumoniae*, that promotes carbapenem resistance spreading among unrelated species [8,88].

MGEs also play a role in the creation of “resistance islands,” where different ARGs come together on plasmids, integrative and conjugative elements (ICEs), or genomic islands. This gives rise to the development of MDR and XDR plasmids in lineages found globally [13,62].

Another key mechanism is the stepwise capture and assembly of ARGs through transposons and integrons, generating mosaic MGEs that often carry resistance, virulence, and fitness genes. *IncX3* plasmids harboring *bla*_NDM_, *qnr*, and *mcr* exemplify such “superbug plasmids” [8]. Importantly, MGEs facilitate interspecies and inter-ecosystem transfer, enabling ARGs such as *mcr-1* to move from livestock to humans and establishing One Health reservoirs in soil, water, and wastewater [7,89].

In addition to dissemination, MGEs stabilize ARGs within host populations via maintenance systems such as toxin–antitoxin modules, ensuring persistence even in the absence of selective antibiotic pressure [8,88]. They also package resistance with virulence determinants, producing high-risk multidrug-resistant and virulent clones [88,90]. Furthermore, recombination and rearrangements within MGEs accelerate ARG evolution, creating novel resistance alleles and serving as “innovation hubs” [8,88]. Finally, epidemic plasmid backbones such as *IncF*, *IncI*, and *IncX* enable ARGs to spread more rapidly than clonal pathogens, contributing significantly to global outbreaks [13].

## 5. Evolutionary Ecology and Selection Pressures

### 5.1. Ecological and Evolutionary Drivers

The ecological evolution of MGEs is a critical factor for explaining the persistence and dissemination of ARGs in nature. MGEs such as plasmids, transposons, integrons and bacteriophages are the part of bacterial mobilome through which genetic exchange takes place and are implicated in the reciprocity between evolutionary events taking place at the microbial level with ecosystem-level features [12].

In microbiomes, mobile genetic elements are consistently conserved due to selective pressures that favor the retention of these elements, such as their role as vectors for resistance to antibiotics, heavy metals, disinfectants, or other “environmentally stressed” conditions [91]. These conditions create a wetting scenario that influences the corresponding mobile horizontal transfer, even at sub-inhibitory concentrations of antibiotics, due to the partial retention of MGEs carrying resistance or metabolic adaptive genes. MGEs not only carry antibiotic resistance genes, but their surrounding ecological context can also confer a fitness advantage to the host. The dynamic interplay between environmental pressures and genetic mobility drives the evolutionary adaptation of both the host and the MGEs, enabling their stable long-term coexistence [92].

There is an evolutionary balance between the cost of carrying and fitness advantage to maintain MGEs in the chromosome. Although the acquisition of plasmids may place metabolic costs on their hosts, compensatory evolution in either the plasmid or host genome often serves to reduce such cost over time and stabilize coexistence [93]. Therefore, these MGEs can become an integral part of the bacterial lineages even when the selective forces which led to their acquisition have relaxed. This evolutionary stabilizing is the reason for antibiotic resistance remaining in microbial populations even when use of antibiotics has been significantly decreased or stopped [94].

The ecological and evolutionary drivers of MGEs are therefore central to understanding the durability of the global resistome. Environments such as soil, wastewater, and animal microbiomes function as reservoirs and mixing zones for MGEs, facilitating continuous gene exchange and adaptation [95]. Recognizing these interconnected processes is essential for developing strategies that address resistance evolution at its source linking antimicrobial stewardship with environmental management and microbial ecology under the One Health framework.

### 5.2. Antibiotic Selection

The strongest driver for resistance that links to the evolutionary dynamics and stability of MGEs carrying ARGs is the use of antibiotics therapeutically or prophylactically, and accidental exposure. The introduction of antibiotics in the practice of the human, veterinary and agricultural sector has led to profound alterations of microbiota with accessory resistance genes changing by becoming part of the core genome. Uncontrolled use of antimicrobials in health and beyond enhances the magnitude of this selection, so that antimicrobial resistance has now turned into a continuously running evolutionary force favoring the simultaneous occurrence of MGEs among those organisms potentially co-carrying ARGs that can be exchanged by HGT [91,96].

The selective landscape of antibiotics exposure is broad above therapeutic threshold. Antibiotics from pharmaceutical manufacturing, hospitals, agricultural effluents and waste water discharges also find their way to natural systems in sub-inhibitory concentration by environmental pollution [97].

These concentrations are sub-lethal for bacteria, but are very effective in promoting genetic exchange. The sub-inhibitory concentrations lead to an enhanced frequency of conjugation, transformation and transduction events, as well as intensified expression of recombinases and transposases, stress responses like the SOS system increasing mutagenesis and the mobilization of MGEs [76].

The ecological consequences of this are significant. Sub-inhibitory concentrations of antibiotics in terrestrial (soil, sediments) and aquatic environments have also been linked to the enrichment of resistant strains and/or plasmids carrying the ARGs, establishing a persistent reservoir of resistance genes [95]. In agricultural environment, antimicrobials for growth promotion or prophylactic use in livestock also select for resistant bacteria in the gut microbiota and these could end up being released into the environment via manure and wastewater [98]. These bacteria, often linked to conjugative plasmids, are incorporated into wider bacterial communities through the interspecies transfer of ARGs from clinical and non-clinical habitats by MGE.

The experimental proof for the effect of sub-inhibitory antibiotic exposure on HGT has been very well established. Indeed, residues of fluoroquinolone compounds have been reported to enhance plasmid conjugation between *E. coli* populations [99], whereas β-lactam and tetracycline exposures can increase integron recombination and transposon expression in general terms [100]. Thus, selective pressure of antimicrobials is fatal and thus emphasizes the presence of antimicrobial residues in the environment as a potent evolutionary factor shaping mobilome.

### 5.3. Co-Selection by Heavy Metals and Biocides

Co-selection by means of heavy metals and biocides is one of the most important drivers for the maintenance and spread of AMR. The use of antibiotics in agriculture, aquaculture, industrial activities, and healthcare can contribute to the formation of environmental reservoirs where resistant bacteria survive even in the absence of antibiotics [101]. Genetic association between metal and biocide resistance genes and also with ARGs occurs when these are located on the same MGEs (plasmids, transposons and integrons), promoting co-selection, their simultaneous transfer to bacteria or stable acquisition by bacterial populations [102].

Heavy metals, such as copper (Cu), zinc (Zn), arsenic (As), mercury (Hg) and cadmium (Cd), are widely utilized feed additives, fertilizers and disinfectants in animal husbandry and crop development under modern agricultural practice. Since these metals are nondegradable, they accumulate in soil and water systems where they constitute a strong sustained selective force on microbial populations. A number of investigations have proven the positive correlations between the metal resistance operons (e.g., *czc*, *ars* and *mer*) and ARGs (e.g., *bla*_CTX-M_, *tet* and *sul*) on the same plasmids or integrons. For example, Enterobacteriaceae isolated from pig farms that utilize high copper feed supplements commonly harbor co-sited Cu and antibiotic resistance genes, demonstrating the indirect toll of agricultural metal use on antibiotic resistance maintenance [103].

Likewise, biocides (such as Quaternary Ammonium Compounds, triclosan and chlorhexidine) serve as sanitizers and preservatives in hospitals, households and food processing facilities. Exponential exposure to sub-lethal concentrations of these compounds may promote the bacterial population of drug efflux pump-related genes like *acrAB-tolC* and *mexAB-oprM* that mediate cross-resistances to multiple antibiotics [104]. The genetic proximity of *qacEΔ1* and class 1 integrons, which are frequently associated with β-lactamase or sulfonamide resistance genes, demonstrates how biocide selection can preserve and spread clinically relevant ARGs in non-clinical reservoirs [105].

Metagenomic investigations of sediments and agrosoils have proved the relationship between metal contamination, and ARGs and MGE distribution. These data demonstrate that co-selection does not necessarily correlate with the direct presence of antibiotics, but metals and biocides represent additive selection forces for R-gene maintenance and dissemination in the bacterial population/ecological niche [89].

The implications of co-selection are far-reaching with regard to the management of AMR. Persistent pollutant heavy metals and biocides are maintaining a long-term background selective pressure that can erode antibiotic stewardship. As such, successful control of AMR will require One Health-led interventions to consider co-selective pressures within the environment, agriculture and industry in order to regulate the use of metals and biocides, as well as antimicrobials, with a view to limiting resistance globally [106].

### 5.4. Fitness Costs vs. Compensatory Evolution

Mobile genetic elements, including plasmids, transposons and integrons, are frequently characterized by a fitness cost to the bacterial host at acquisition or persistence into bacterial populations. This is due to the metabolic cost of replicating extra DNA, expressing resistance genes and maintaining machinery for horizontal gene transfer [92]. Nevertheless, MGEs are maintained and can even be stably inherited despite this drawback. This enigmatic phenomenon is attributed to compensatory evolution, where beneficial mutations in the host chromosome or plasmid revert fitness costs while maintaining resistance [93].

Experimental evolution experiments have also demonstrated that the strength of plasmid-associated fitness costs differs among plasmids and between copy numbers within a given plasmid (Carroll & Wong, 2018 [107]). Plasmids with broad host-range (such as *IncP-1*) typically carry an initial higher cost to cells owing to their larger size and extensive conjugation machinery, whereas smaller-host-range plasmid subtypes (like *IncF*) can be more easily integrated into individual bacterial lineages [108]. In the course of several generations, bacteria with expensive plasmids can accumulate beneficial mutations in chromosomal loci that are implicated in replication, transcription or stress responses such as *rpoB*, *dnaA* or global regulators like *arcA*, which compensate for some of the burden due to the plasmid and restore growth rates close to those observed for isogenic plasmid-free strains. Likewise, plasmid-borne mutations in replication or maintenance loci can diminish the cost of expression, which stabilizes the host–plasmid association [92].

One well-documented example is *IncF* plasmids in *E. coli* that may have an initial fitness cost (e.g., a growth defect and reduced competitiveness) but restore cost-neutrality by compensatory chromosomal mutations or co-evolutionary changes in the control of plasmid replication [109]. When stabilized in this fashion, plasmids can exist without selection pressure for resistance genes as long-term repositories of them. This is a good way to make MGEs much more than just transients’ vectors, a coordinated part of bacterial genomes and the long-term persistence of AMR in populations and environments [93].

Importantly, the balance between costs of fitness and compensatory evolution or environmental factors decides the epidemiological success of resistance plasmids. Plasmids harboring *bla*_CTX-M_ or *mcr-1* spread continuously in the world at low fitness cost, as demonstrated by plasmid relatedness and epidemiological settings [23]. In addition, costly plasmids must also be able to introduce themselves and persist in habitats experiencing intense selection such as hospitals or farms where antimicrobials and metals are commonly used [110]. This balance shows that the engine of resistant determinant maintenance and global dissemination is the evolutionary fit between plasmid and host expressed in its optimality.

### 5.5. Plasmid Ecology

They are very likely the most flexible elements of the bacterial mobilome and play critical roles in the ecology and evolution of AMR. The fact that plasmids persist and circulate in the long term is determined by selective forces but also by some of the innate characteristics that make up these factors, like host-range, conjugation efficiency and stability mechanisms, which assure successful plasmid ecology within microbial communities [23]. Knowledge of the ecology of plasmids is vital to understanding how resistance genes are being mobilized and disseminated among diverse habitats, including clinical settings and natural environments.

#### 5.5.1. Host-Range and Transfer Dynamics

At the core of every plasmid–host-range security system, there is its own host-range which is the most important and direct contributor to the taxonomic breadth of the host-range of bacteria in which they can replicate until being lost. Plasmids belonging to the IncP and IncA/C groups are considered broad host-range (BHR) plasmids capable of replicating across diverse bacterial taxa (capable of replication across multiple taxonomic groups (e.g., IncP, IncA/C)) [111]. Narrow-host plasmids such as those *IncF* and *IncI* types are commonly confined to Enterobacteriaceae but may also be endemo-epidemic in some clonal populations due to being highly replicative (plasmids with high copy number (>10–50 copies per cell)) and having maintenance systems. This diversity influences the level of spatial and taxonomical structuring of resistance flow [112].

Plasmid success is also highly dependent on conjugation rates. Highly conjugative plasmids, such as *IncP* and *IncN*, exhibit high promiscuity and spread quickly in the absence of selection pressure for antibiotic resistance, whereas low-transfer plasmids depend more on vertical inheritance and stable maintenance [108]. Environmental parameters, namely temperature, nutrients and microbial abundance, also modulate conjugation frequencies influencing plasmid persistence in wastewater, soil and host-microbiomes [113].

#### 5.5.2. Stability and Maintenance Systems

Plasmids utilize various mechanisms to promote their maintenance in bacterial populations, even in the absence of selection. Among them, toxin–antitoxin (TA) systems and partitioning systems play a significant role as genetic modules contributing to the plasmid stability. TA systems (e.g., *hok*/*sok*, *ccdAB* and *pemIK*) operate through post-segregational killing: daughter cells losing the plasmid die because the more stable toxin outlives its fragile antitoxin [114]. A well-regulated plasmid partition system (*parAB*/*parMRC*) leads to correct segregation of plasmids at cell division, lowering the probability that a plasmid becomes lost in a dividing population [24]. These mechanisms translate plasmid carriage to an essentially irreversible phenotype, promoting their extended live-long day.

Furthermore, the fitness cost is minimized, and the plasmid–host interactions are stabilized by maintenance genes and addiction modules or multimer resolution systems that are frequently present in plasmids. These characteristics are particularly favorable within structured habitats, such as biofilms, manure, and the slurry environment itself, where close cell proximity facilitates conjugation and maintenance. These are “gene trading centers” in which plasmids carrying AMR genes can be exchanged between commensals, pathogens and environmental flora [115].

#### 5.5.3. Ecological Niches and One Health Implications

The ecological success of plasmids is being experienced in the increasingly linked One Health settings. Within hospitals, plasmids carrying *bla*_KPC_, *bla*_NDM_ or *mcr-1* can lead to multidrug-resistant outbreaks within Enterobacteriaceae [112]. In agrarian contexts, plasmids such as *IncQ* and *IncA*/*C* have been identified in animal manure and soil microbiota associated with agricultural antibiotic use and metal usage to ARG persistence [113]. Water environments are hotspots for the exchange of plasmid, because the human and animal microbial communities intersect at wastewater treatment plants [116].

### 5.6. Environmental Reservoirs

The environment is a key reservoir of the transmission and spread of MGEs, multidrug-resistant bacteria and ARGs. In contrast to the clinical environment, in which exposure to antibiotics is usually direct and intense, environmental reservoirs are complex ecosystems where multiple selective pressures come into play. Environmental microbiomes, including soil and marine ecosystems, harbor what is referred to as the “ancient resistome,” representing naturally occurring resistance genes that predate clinical antibiotic use [91].

Soil microbiomes contain what is referred to as the “ancient resistome”: naturally occurring β-lactamase and tetracycline resistance determinants that evolved prior to the modern usage of antibiotics [117]. Metagenomic analysis of permafrost and remote arctic soils showed ARG sequences almost identical to modern resistance genes, suggesting high evolutionary conservation [118]. While these genes developed as a response to natural antibiotics produced by soil microorganisms, the exploitation of the anthropogenic activities like agricultural antibiotic use and manure application have sped up their mobilization into clinical bacterial species through plasmids, integrons and transposons [119].

Wastewater treatment plants (WWTPs) are also identified as increasingly important hotspots of the environment for MGE-facilitated resistance transfer. In aggregating runoffs from the hospitals, households and agriculture, dense microbial communities dominated by various pathogenic antibiotic resistant organisms co-habit within these systems often with closely scheduled periods for conjugation events and the transformation of plasmid [120]. Sub-lethal levels of antibiotics, disinfectants and heavy metals in WWTPs further increase selective pressure for the maintenance of plasmids as well as allowing ARG transfer between Enterobacteriaceae, *Pseudomonas*, and *Acinetobacter* spp. [121]. Recently, metagenomic studies have revealed that class 1 integrons, *IncP-1* plasmids and transposons harboring *bla*_CTX-M_ and *sul1* genes are prevalent in activated sludge and effluent discharges, indicating the ongoing gene transfer circulation from environmental bacteria to clinical isolates [122].

There are additional ecological reservoirs of MGE-mediated ARG persistence in animal farms and aquaculture systems. The widespread application of antibiotics and also metals like zinc and copper in feed additives will apply strong co-selection to propagate resistant bacteria [123]. Transferable *mcr-1*-, *tet(X4)*-, and *bla*_NDM_-bearing plasmids have been isolated from poultry and fish farms, suggesting these sites act as amplifiers for MDR [31]. Runoff from these facilities may facilitate the introduction of ARGs into adjacent soils and water systems, allowing for recombination between environmental microbiota, thereby increasing the gene pool available to pathogens.

Recently, the airborne transmission of ARGs has become an added dimension of concern. Aerosolized bacteria along with extracellular DNA fragments, carrying ARGs and MGEs, have been found inside hospital ventilation systems, urban air and animal housing facilities [124]. These findings broaden the concept of ‘‘One Health” theory by identifying the air microbiome as one more pathway for ARG transmission.

### 5.7. Role of Biofilms in ARGs Dissemination

Biofilms are increasingly recognized as critical “hotspots” for the dissemination of antibiotic resistance genes (ARGs) due to their unique structural and ecological characteristics [54]. Within biofilms, dense microbial communities are embedded in an extracellular polymeric substance (EPS) matrix that facilitates close cell-to-cell contact, thereby significantly enhancing horizontal gene transfer (HGT), particularly conjugation and natural transformation [110]. The EPS matrix also promotes the retention of extracellular DNA, further supporting genetic exchange. Importantly, biofilms confer protection against antimicrobial agents, creating sub-inhibitory antibiotic gradients that intensify selective pressure and stimulate the activation of MGEs, including plasmids, integrons, and transposons [71]. Consequently, ARGs are often enriched and persist longer within biofilm-associated populations compared to planktonic cells. Emerging evidence highlights environmental biofilms, particularly those forming on marine plastics (the “plastisphere”), as novel reservoirs facilitating the accumulation and global dissemination of multidrug-resistant bacteria [54,72]. In clinical settings, biofilms on medical devices such as catheters and chronic wound surfaces serve as persistent sources of resistant infections, further emphasizing their central role in the epidemiology of antimicrobial resistance [8].

## 6. Epidemiology—Case Studies Linking MGEs to Clinical Outbreaks

The dissemination of AMR through MGEs is arguably one of the most major evolutionary and epidemiologic challenges for microbiology today. MGEs, the molecular machines that plasmids, transposons, integrons and insertion sequences facilitate by their intrinsic nature. The natural co-selection of resistance gene transfers in between genetically unrelated bacterial species, colonizing numerous ecological niches. This mobility may have an impact in turning individual local resistance events into regional or global public health threats (Table 4) [112].

These surveys spanning two decades are a testimony that many of the severest cases of AMR have been caused by single MGEs harboring significant ARGs. The plasmid-mediated dispersion of *bla*_NDM-1_, *mcr-1* and *tet(X4)* genes and transposon carrying *bla*_KPC_ are also characteristic of MDR organisms from mobile genetic elements. It is not just in the clinical environment where MGE is important. Resistance genes that formerly were restricted to the hospital setting have now become broadly distributed across livestock, aquaculture waste water and environmental strains, evidencing their One Health role [95].

### 6.1. Plasmid-Borne bla_NDM-1_ Dissemination

The emergence of the New Delhi metallo-β-lactamase-1 (*bla*_NDM-1_) gene from *K. pneumoniae* and *E. coli* in India 2008 was a turning point towards the global epidemiology of carbapenem resistance. Unlike the prior carbapenemase genes, which had been confined to specific lineages, *bla*_NDM-1_ was rapidly mobilized among a variety of plasmid backbones that allowed for its intercontinental spread in just a few years. The gene has subsequently been detected in several other bacterial hosts including *Enterobacter*, *Acinetobacter*, *Pseudomonas* and *Citrobacter* [126].

*bla*_NDM-1_ is most commonly situated in broad host-range plasmids such as *IncA*/*C*, *IncFII*, *IncX3*, and *IncHI1*. These plasmids often also contain other resistance genes, against the vast majority of antibiotics, e.g., genes *bla*_CTX-M_, *armA* and *qnr* [133]. Comparative genomics also demonstrated that *bla_NDM-1_* is frequently integrated into an insertion sequence, ISAba125, that facilitates its transposition to new plasmid backgrounds and enhances its potential for horizontal transfer [138].

Having originally emerged in the Indian subcontinent, a huge dissemination of *bla*_NDM-1_ occurred there due to international visiting, medical tourism and environmental infection. Initial reports of NDM producers have been observed in patients previously admitted to hospital in South Asia, UK, Canada and Middle East [126]. Other studies subsequently demonstrated that *bla*_NDM-1_ was not confined to the hospital setting. It has been found in sewage, surface water and drinking water, indicating environmental contamination as a source of possible secondary transmission [125].

The control, prevention and broader public health aspects of the One Health figuration of *bla*_NDM-1_ dispersal are among the most pressing challenges. The gene has been detected from animal sources including poultry, cattle and pets, as well as from food sources and the environmental reservoir. It is a cross-sector distribution network, which means NDM-positive bacteria are exchanging between humans and animals and the environment based on conflicting ecological selection forces and inadequate wastewater treatment. For instance, investigations in China found a high prevalence of *bla*_NDM-1_ among *Enterobacter cloacae* isolates originating from a hospital or river water specimens, indicating the constant movement of resistance genes across hospitals and the environment [139].

### 6.2. Plasmid-Mediated mcr-1 and Colistin Resistance

The worldwide detection of mobilized colistin resistance (*mcr*) genes, especially *mcr-1*, has revolutionized previous knowledge on last-line antibiotic resistance mechanisms. Originally detected in 2015 among *E. coli* from pigs, raw meat, and humans in China, *mcr-1* is responsible for coding an enzyme, phospho-ethanolamine transferase, one that modifies lipid in the bacterial outer membrane leading to a reduction in colistin binding [128]. This finding was the first independent confirmation that colistin resistance could be transferred through plasmids, in contrast to previously believed notions that polymyxins resistance would mainly have a chromosomal localization [140].

The plasmid ecology of *mcr-1* has made a substantial contribution to *mcr-1*’s swift dissemination worldwide. The gene has been identified in different self-transmissible plasmid replicon types, including *IncI2*, *IncX4*, *IncHI2* and *IncP* (which are frequently connected with other multidrug resistance determinants such as *bla*_CTX-M_, *qnrS* and *floR*) [127]. Plasmid sequencing of comparative isolates suggests that *IncI2* and *IncX4 mcr-1*-positive plasmids have a highly conserved backbone in human, livestock, and food isolates, illustrating the transfer of genes between species and sectors rather than clonal spread.

After its identification, *mcr-1* was found to be present across all continents in clinical, agricultural, and environmental reservoirs and the colonization of humans with ESBL-producing bacteria; this demonstrates the global use of colistin as a growth promoter before regulatory actions. Its presence in production systems in poultry and pigs had already been reported by surveys in Europe, Africa, and South America and parallel surveys detected *mcr-1* among human clinical isolates without livestock exposure [141]. This pattern highlights the influence of three food chains and environmental contamination on the transfer of mcr-positive bacteria among ecosystems.

Environmental monitoring has also shown that the *mcr-1* genes can be widely detected in wastewater treatment facilities, river sediments and retail vegetables; they indicate a continuous loop of contamination and re-entry to human community. *mcr-1*-positive *E. coli* from fresh vegetables in Guangzhou and clinical isolates are being detected in the same ecological region of Guangdong, which could represent a farm-to-fork transmission route. Additionally, wildlife such as migrating birds has also been reported to harbor *mcr*-positive Enterobacteriaceae that can serve a potential vehicle for worldwide distribution in the environment [142].

The discovery of novel *mcr* variants (mcr-2 to -10) in different genera indicates that colistin resistance is not a monogenic feature but an expandable mobilome [31]. Notably, *mcr-3* and *mcr-9* have been linked to easily conjugative *IncHI2* plasmids, challenging efforts for containment. The enhanced genetic diversity and the linked persistence of *mcr*-bearing plasmids, even without selection, justify the continued sustenance of colistin throughout the global resistome.

Control measures today include a One Health approach to antimicrobial stewardship, genomic surveillance and agricultural regulation. For instance, the *mcr-1* prevalence in humans and animals dramatically decreased within 2 years after China prohibited colistin use as a feed additive, suggesting implemented policy practices were an effective way to control the spreading [143]. Continued genomic surveillance at the human, animal and environmental interface is essential to assess adaptive trajectories of resistance mediated by mcr.

### 6.3. Plasmid-Mediated tet(X3/X4) Genes for Tigecycline Resistance

The recent discovery of plasmid-mediated *tet(X)* variants has prompted considerable attention within the AMR field, as these genes compromise tigecycline, a glycylcycline and often the last line of defense against multi-resistant Gram-negative pathogens. In contrast to conventional *tet* genes that are responsible for the exportation or ribosomal safeguarding, *tet(X)* encodes a flavin-dependent monooxygenase that can enzymatically hydrolyze tigecycline and thus nullifies its effectiveness [130].

The *tet(X3)* and *tet(X4)* alleles were initially described in *E. coli* and *Acinetobacter* isolates from a swine farm and environmental samples of China in 2019. The two genes were found on self-transferable plasmids, mainly belonging to incompatibility groups *IncQ1*, *IncFII* and *IncX1* and frequently associated with insertion sequences (ISCR2), mediating their spreading. Unique to *tet(X4)* is that it had a wide host-range and high conjugative frequencies, facilitating its spread among Enterobacteriaceae and *Acinetobacter* spp. [130].

Molecular epidemiological investigations have demonstrated that *tet(X4)* plasmids also carry other resistance genes, including *floR*, *sul2* and *bla*_CTX-M_ variants. This dual resistance mechanism plays a positive role in both the stability and transmission of *tet(X4)* within tigecycline-abstain-like hospital populations. In addition, as *tet(X)* genes are always carried along with *mcr* and *bla*_NDM_ by hybrid plasmids which could be horizontally transmitted, we have to confront the risk of stepping into a “pan-drug” resistance period in Enterobacteriaceae. *EcoTet(X)* has been identified from environmental water, manure and soil sample near a livestock farm using the environmental surveillance method, implying the over-spillover of resistance genes [130]. These results highlight the need for effective intergraded genomic surveillance systems and the prudent use of antimicrobials in both human and veterinary practices.

*Tet(X3*/*X4)* dissemination is of great clinical concern. Tigecycline is one of the last options to treat patients infected by carbapenemase producers, and its acquisition through mobile elements constitutes a direct hit to our available armamentarium. The fitness neutrality of *tet(X)* in bacterial populations and the absence of an overt antibiotic-selective cost suggests that its spread could not be effectively contained and it might be incorporated to be a stable part of the global resistome.

### 6.4. Dissemination of bla_KPC_ via Tn4401: Transposon–Plasmid Synergy in Global Outbreaks

The *K. pneumoniae* carbapenemase (KPC) is one of the most clinically important β-lactamases from the past 20 years. This enzyme is encoded by the gene *bla*_KPC_, which was initially discovered in *K. pneumoniae* strains and later found to be a predominant cause of carbapenem resistance among hospital-acquired infections globally [132]. The worldwide spread of *bla*_KPC_ reflects not only clonal dissemination among high-risk bacterial lineages, but also its successful mobilization by the Tn4401 transposon, facilitating horizontal transmission on various plasmid backbones [131].

Tn4401 is a 10 kb composite transposon with integral inverted repeats (IRs) that belongs to the Tn3 family; it carries a pair of genes encoding for its movement, *tnpA* and *tnpR*. Several isoforms (Tn4401a–f) have been described, with minor variants including short deletions or insertions up-stream of *bla*_KPC_ that may influence gene expression levels and possibly the resistance phenotype [144]. *bla*_KPC_ can therefore transpose between plasmids, predominantly *IncFII*, *IncN*, and *IncL* incompatibility groups, enabling its propagation among Enterobacterales populations [131].

From an epidemiological perspective, *Tn4401*-based *bla*_KPC_ has played a central role in hospital outbreaks on several continents, including North America and South America as well as Europe and Asia. In the US, *K. pneumoniae* ST258 has emerged as a predominant clone carrying *bla*_KPC-2_ and *bla-3* variants associated with endemic hospital-wide spread [145].

Environmental stability and inter-specific transfer add to the problems of control. *bla*_KPC_-positive *Enterobacter*, *Citrobacter* and *Pseudomonas* isolates have been reported from wastewaters and hospital effluents, indicating that these elements are also outside of patient populations. The observation of identical Tn4401 cassettes on unrelated plasmids in different species indicates repeated transposon capture events, meaning that carbapenem resistance can spread by different clonal backgrounds [146].

### 6.5. Resistance Islands and Clonal Expansion

Long-term genetic stability depends on chromosomally integrated resistance islands, which contribute to the continued and epidemically relevant spread of antimicrobial-resistant bacteria. Intergenic islands, also known as resistance islands, represent such large genomic areas and serve as the recipients of several ARGs that often harbor insertion sequences (ISs) and integrons, enabling their further evolution. These include the AbaR islands in *A. baumannii*, the SGI1 elements in *S. enterica* and complex resistance regions identified within epidemic *K. pneumoniae* clones such as ST258 [28,147].

One of the most well-studied chromosomal platforms conferring multidrug resistance in *A. baumannii* is the AbaR resistance islands. These islands project in size from 19 to over 100 kb and with comM being a common integration site, they harbor an array of determinants for resistance to aminoglycosides, β-lactams, tetracyclines and sulfonamides. The mosaic nature of these islets shows a pattern consistent with multiple acquisitions promoted by class 1 integrons and IS26 elements, which facilitate intra- and inter-island recombination. The high prevalence of AbaR in international clones, including IC1 and IC2, illustrates its contribution to the worldwide spread of multidrug-resistant *A. baumannii* in the hospital setting [28].

Likewise, the *Salmonella* genomic island 1 (SGI1) has facilitated the development of epidemic multidrug-resistant *S. enterica* serovars worldwide, including *S.* Typhimurium DT104. The integration of SGI1 is to the chromosomal *trmE* site and it carries a complex integron structure encoding ampicillin, chlorampheniocol, streptomycin, sulfonamide and tetracycline resistance [147]. Since its discovery in the 1990s, variants such as SGI1-K and SGI1-L have been found among different serovars and even strains of *Proteus mirabilis*, indicating interspecies lateral gene transfer [148]. The success of SGI1 illustrates how stable chromosomal inserts can provide long-term vehicles for resistance determinants with minimal impact on fitness in epidemic clones.

The clonal expansion hypothesis serves to co-ordinate the significance of MGEs and to focus on the evolutionary success of particular bacterial lineages that have incorporated resistance determinants into a stable genomic background. The clones of *K. pneumoniae* belonging to ST258 and its SLVs (e.g., ST512) have spread worldwide, the result of fusion events between chromosomal resistance islands and other plasmid-encoded carbapenemase genes, including *bla*_KPC_ [149]. To use international *A. baumannii* clones I and II as well as *E. coli* ST131 as examples, when resistance islands become genomically fixed, clonal spread can carry their epidemiological effects much further than would be possible through the mechanism of HGT alone [146].

### 6.6. One Health Case Studies

The horizontal transfer of AMR genes via MGEs has outstripped classical clinical silos, and this further emphasizes the intimate relationships between man, animals and environment. An emerging line of evidence that supports the One Health perspective reveals that resistance genes and their MGE carriers move bidirectionally between these domains, and in so doing make containment and surveillance more problematic [95].

The global distribution of *bla*_NDM_ is an epiphenomenon that has moved on from other backgrounds around the world and that encodes the New Delhi metallo-β-lactamase (NDM). A wonderful example of cross-fertilization is the international movement, over a time period, of this gene. First found in India in isolates of *K. pneumoniae* and *E. coli* from a patient in 2008, *bla*_NDM-1_ has been reported to be present across an astounding variety of habitats, including hospital effluents, wastewater, rivers and wildlife [125]. *bla*_NDM-1_-positive Enterobacteriaceae have been found in river sediments and migratory birds in China and Europe, suggesting that not only human activities but also animal carriers contribute to the high environmental persistence of this gene [142]. Together, these findings indicate that clinical containment might successfully limit its spread but environmental resistance reservoirs could feed a long-lasting gene flow back to the human populations.

Another major inter-domain transfer is the *mcr-1* gene, that places livestock and human beings who have used its direct precursor, colistin, a last-resort antibiotic for both animals and people. It is a gene that was first described in 2015 for *E. coli* isolates from pigs but has spread to retail meat, vegetables and clinical human isolates around the world within a few years of its first report [128]. Genomic comparison indicated *mcr-1* is predominantly located in conjugative plasmids belonging to the *IncI2* and *IncX4* compatibility groups, with >99% sequence identity on their backbone among isolates from food animals, human and environmental samples [127]. It implies that the plasmids are the modes of transmission, not the clonal spread of bacteria. Consequently, the widespread use of colistin in animals has been described as a major form of selective pressure contributing to the dissemination of *mcr-1* throughout ecosystems.

Other selective pressures maintain the resistance outside of clinical reservoirs, beyond antibiotic usage. Colistin-resistant *E. coli* and carbapenemase-producing *Klebsiella* have been detected in migrant birds on transcontinental flyways and rivers that receive wastewater from urban and agricultural sources [150]. Furthermore, the identical plasmid backbones harboring *bla*_NDM_, *mcr-1* and *qnrS* could be found in hospital, farm and aquaculture environments, additionally supporting the existence of a shared mobilome that circulates among different ecological hosts [151].

## 7. Detection, Monitoring, and Genomic Surveillance Methods

A range of laboratory and genomic approaches is used for AMR detection and monitoring to identify, characterize, and track resistance determinants (Table 5). Conventional laboratory methods that can help in understanding mobile genetic elements in the transfer of resistance genes include conjugation assays, PCR replicon typing, plasmid profiling, etc. These previous methods could be complemented with newly developed approaches, such as databases and sequencing technologies, including whole genome sequencing and targeted plasmidomics, which are now available and provide a greater level of resolution and accuracy to support surveillance and overcome the limitations of previous methods.

## 8. Public Health Impact and Clinical Implications

### 8.1. Impact on Treatment and Clinical Outcomes

AMR is currently one of the largest threats to global public health [12,90,166,167]. A major example of the clinical threat that MGEs pose is carbapenem-resistant Enterobacterales (CRE). Plasmids that carry a variety of resistance determinants like *bla*_KPC_ and *bla*_NDM_ can spread rapidly in healthcare environments, making carbapenems, the last line of treatment, ineffective. Clinicians are left to use toxic or less effective alternatives, like polymyxins [168].

When multiple MGEs carrying distinct resistance determinants co-occur, they synergistically exacerbate MDR, often rendering empirical treatment ineffective [90]. When more than one MGE with different resistance determinants co-occur, the synergy contributes to even more multidrug resistance than either MGE alone, and may even render empirical treatment ineffective [90]. Multidrug-resistant pathogens can lead to delays in the initiation of appropriate antimicrobial therapy, which may lead to increased mortality, longer length of hospital stay, and increased costs to the healthcare system [169], and expose patients to more risks, such as secondary infections. Numerous clinically important bacteria, notably the ESKAPE bacteria (*Enterococcus faecium*, *Staphylococcus aureus*, *Klebsiella pneumoniae*, *Acinetobacter baumannii*, *Pseudomonas aeruginosa*, *Escherichia coli*, and *Enterobacter* spp.), have been increasingly linked to drug-resistant infections. These pathogens are particularly hard to treat due to their possession of antimicrobial resistance genes, acquired via horizontal gene transfer, most often mediated by various mobile genetic elements [8,88,170,171].

### 8.2. Infection Control and Antimicrobial Stewardship

Infection control (IC) and antimicrobial stewardship (AMS) are related processes impacting infection management, controlling the emergence of antimicrobial resistance and the optimization of patient outcomes. Effective IC protocols, including hand hygiene, environmental cleaning, patient isolation, and active surveillance, are necessary to interrupt routes of transmission in healthcare settings [172]. Infection prevention and control (IPC) is a significant action based on evidence that seeks to protect patients and healthcare providers against preventable infections, even infections caused by drug-resistant organisms, and actively engages physicians, nurses, pharmacy practice, and professionals [173]. Antimicrobial stewardship (AMS) is a collaborative effort with the key objectives of enhancing patient outcomes, limiting the development of antimicrobial resistance, and reducing unnecessary costs [174]. Antibiotic stewardship programs (ASPs) are critical components of addressing antibiotic resistance worldwide. By promoting the selection of the most effective antibiotic, dose, duration of therapy, and route of delivery, AMS and antibiotic stewardship programs may also help to reduce the incidence of adverse effects and costs [175,176]. Although it is essential in effective AMS programs to take MGE dynamics into account for optimal antibiotic selection and to limit antibiotic resistance gene transfer, it is even more important with respect to the case of hospital-acquired infection [177]. Integrated infection control–antimicrobial stewardship programs have demonstrated meaningful reductions in the use of antimicrobial agents, in the incidence of multidrug-resistant organisms, and in the rates of healthcare-associated infections [178].

### 8.3. Economic and Societal Impact

The worldwide distribution of antibiotic resistance is largely due to mobile genetic elements (MGEs) [167]. MGE-mediated antimicrobial resistance has far-reaching and complex social and economic impacts. AMR was among the leading causes of death worldwide in 2019, with an estimated 1.27 million deaths directly attributable to AMR and a further 4.95 million deaths indirectly attributable to AMR [179]. In addition to its health and mortality effects, AMR places a tremendous economic strain, with an estimated excess cost to health services of US$1 trillion by 2050, and an estimated drag on global GDP of US$1–3.4 trillion per year by 2030 [1]. Antimicrobial resistance has implications beyond health systems, impacting agricultural production, aquaculture and livestock, resulting in trade and productivity constraints that are especially burdensome for low- and middle-income countries. Further, resistance genes spread environmentally via wastewater and soil, leading to community exposure, which emphasizes the need for a One Health response to AMR [180].

## 9. Control Strategies: From Prevention to MGE-Targeted Interventions

### 9.1. Conventional Interventions

In the case of diminishing both selection pressure and the chances for HGT, an evidence-based approach to conventional interventions plays a fundamental role in the acquisition and dissemination of ARGs (Table 6) [60,181]. Guideline-based prescribing, veterinary stewardship, limitation of last-resort medicines, and audit-and-feedback mechanisms, known as rational antimicrobial use, are necessary to reduce the MGE-mediated transmission. The World Health Organization’s Global Action Plan, as well as international reviews, make this case unequivocally [166].

Infection prevention and control (IPC) in healthcare and veterinary settings are essential for interrupting transmission pathways and reducing bacterial loads, thereby effectively curtailing the potential for plasmid- and ICE-mediated outbreaks driven by horizontal gene transfer [60,182]. A previous study shows that plasmid surveillance with routine IPC, including mapping outbreak plasmids and high-risk clones via replicon typing and long-read sequencing, improves outbreak resolution and targeted interventions in NDM-5-producing *Enterobacterales* outbreaks [183].

Effective management of wastewater and the broader environment constitute a critical, yet frequently overlooked, aspect of conventional strategies to control antimicrobial resistance. Wastewater treatment plants function both as reservoirs for ARGs and mobile genetic elements and as potential conduits for their release into natural ecosystems [106,184]. Enhancing treatment trains through improved biological processes, advanced oxidation, UV irradiation, and targeted adsorption alongside implementing steps that minimize viable host bacteria and free DNA, can significantly reduce the release of antibiotic resistance genes into the surrounding environment [185]. Meta reviews of treatment efficacy and mechanistic studies document substantial variability across technologies and emphasize that environmental engineering must be incorporated into national AMR plans [186].

Finally, policy reforms in agriculture and aquaculture such as limiting non-therapeutic antibiotic use, eliminating the use of critically important drugs for growth promotion, and mitigating co-selective pressures from heavy metals and biocide residues, are essential for decreasing environmental reservoirs and the selective forces that sustain ARG-bearing mobile genetic elements outside clinical contexts. Global One Health guidelines consistently highlight the importance of coordinated regulatory efforts spanning human, animal, and environmental sectors [187,188,189].

### 9.2. MGE-Focused and Novel Strategies

MGEs are one of the main forces behind the worldwide spread of antibiotic resistance. They allow resistance genes to move between different bacterial species, making infections harder to treat [90,167]. In *Acinetobacter baumannii*, a common hospital-acquired pathogen, MGEs such as insertion sequences, transposons, integrons, and plasmids play a key role in resistance to carbapenems and cephalosporins [86,190]. Among these, *ISAba* elements are especially important because their strong promoters increase the production of β-lactamases, enzymes that make the bacteria resistant to many antibiotics [86].

Emerging strategies to control mobile genetic elements focus on the use of conjugation-inhibiting compounds in combination with antibiotics, alongside approaches that consider MGEs themselves as direct therapeutic targets [167]. Environmental approaches to mitigating antimicrobial resistance involve optimized wastewater treatment processes to eliminate both antimicrobial compounds and resistance genes, the application of advanced oxidation methods, and the use of nanomaterials for targeted pathogen removal [190]. These strategies, targeting mobile genetic elements, signify a paradigm shift toward precision antimicrobial interventions, aligning with One Health principles to address the spread of resistance at cellular, community, and global scales [167].

**Figure 4:**
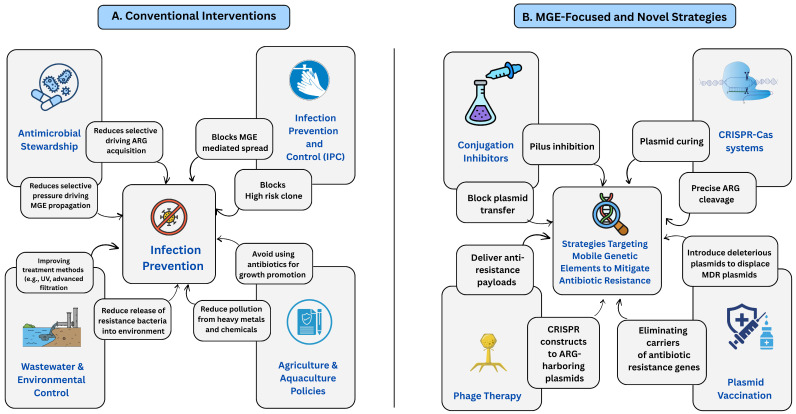
Strategies to mitigate antibiotic resistance through conventional and mobile genetic element (MGE) focused approaches.

CRISPR-Cas systems offer a promising approach for mitigating antimicrobial resistance, acting as RNA-guided adaptive immune mechanisms capable of precisely recognizing and cleaving DNA sequences that encode antibiotic resistance genes (Figure 4) [191]. For curbing HGT and restricting the dissemination of resistance with the help of eliminating plasmids and precisely targeting or directly cleaving antibiotic resistance genes, those systems play a significant role [192]. Complementary approaches to mitigate the dissemination of ARG involve the adoption of advanced oxidation processes, the utilization of nanomaterials for selective pathogen elimination, and the optimization of wastewater treatment systems to enhance the removal efficiency of resistance determinants [190].

Bacteriophages and phage-related particles also make a significant contribution by carrying resistance genes between bacteria as well as offering new therapeutic opportunities. Strategies under investigation to curb the environmental dissemination of antibiotic resistance include the development of conjugation-inhibiting compounds, along with improvements in wastewater treatment, composting practices, and oxidation-based remediation technologies aimed at limiting the spread of resistance through aquatic, terrestrial, and atmospheric pathways [167,190,193].

Serving as pivotal hubs for HGT, by enabling transposition and recombination processes, resistance plasmids facilitate the mobilization of other genetic elements [21]. Control strategies involve implementing a range of interventions, including preventing the airborne spread of ARG, employing nanomaterials for selective pathogen elimination, integrating tertiary wastewater treatment technologies, and adopting advanced oxidation processes to enhance contaminant degradation [190]. Moreover, employing chemical and biological approaches to specifically target plasmids offers a promising strategy for eliminating carriers of antibiotic resistance genes and mitigating the spread of antimicrobial resistance [21].

**Table 6:** Different control strategies for MGE-mediated antibiotic resistance.

Strategy Category	Intervention	Mechanism/MGE Target	Primary Impact (Clinical/Environmental/One Health)	Implementation Status/Readiness	Feasibility/Scalability	Key Notes/Limitations/Ethical Considerations	Reference
Conventional Stewardship	Rational antimicrobial use	Reduces selective pressure driving ARG acquisition and MGE propagation	Clinical: ↓ treatment failures; Environmental: indirect	Widely implemented	High in clinical settings; moderate in veterinary	Requires adherence monitoring; limited effect on environmental ARGs; essential for One Health AMR control	[60,166,181]
	Infection Prevention and Control (IPC)	Blocks clonal and MGE-mediated spread via hygiene, isolation, contact precautions	Clinical: ↓ nosocomial spread; One Health: limits cross-species transmission	Established	High in hospitals/animal facilities	Does not address environmental ARGs; resource-intensive; plasmid surveillance improves outbreak resolution	[60,182,183]
	Wastewater/Environmental Management	ARG and MGE removal via activated sludge, advanced oxidation, UV, and adsorption	Environmental: ↓ ARG reservoir; One Health: limits spread to humans/animals	Implemented in select regions	Moderate–low; infrastructure-dependent	Treatment efficacy varies; environmental engineering should be integrated into national AMR plans	[106,184,186]
	Agricultural and Aquaculture Policies	Restriction of non-therapeutic antimicrobial use; reduce co-selective pressures	Environmental: ↓ ARG amplification; One Health: ↓ transmission to humans	Partial/region-specific	Moderate; policy enforcement challenges	Compliance varies; socio-economic barriers; requires cross-sector regulation	[187,188,189]
MGE-Focused/Novel	Conjugation Inhibitors	Block plasmid transfer via relaxase or pilus inhibition	Clinical and Environmental: ↓ horizontal gene transfer	Preclinical (in vitro/animal models)	Low–medium; early-stage	Specificity and off-target effects under evaluation	[90,167]
	CRISPR-Cas Systems	Plasmid curing, precise ARG cleavage	Clinical: eliminate MDR plasmids; Environmental: ARG reduction	Experimental/proof-of-concept	Low; requires controlled deployment	Biosafety and ecological concerns; precise targeting needed	[191,192]
	Phage Therapy and Engineered Phages	Deliver anti-resistance payloads or CRISPR constructs to ARG-harboring plasmids	Clinical and Environmental: targeted ARG elimination	Preclinical/pilot studies	Low–medium; host-range limitations	Regulatory and ecological challenges; specificity required	[167,190,193]
	Bioremediation Approaches	Microbial consortia, constructed wetlands, advanced oxidation, and nanomaterials	Environmental: reduce ARG reservoir; One Health: limits downstream exposure	Early implementation	Medium; site-specific	Partial ARG removal; integration with wastewater and agriculture required	[190,192]
	Plasmid Vaccination (Speculative)	Introduce deleterious plasmids to displace MDR plasmids	Clinical and Environmental: ARG reduction	Conceptual	Very low; theoretical	Ethical, ecological, and biosafety concerns; hypothetical	[21]
	Conjugation-blocking Probiotics (Emerging)	Engineered commensals expressing pilus/relaxase inhibitors	Gut/Farm microbiomes: ↓ horizontal ARG transfer	Conceptual/experimental	Low	Ecological impact unknown; needs in vivo validation	[167]
	Global MGE Surveillance and Reporting	Real-time plasmid and ARG tracking across humans, animals, and the environment	One Health: early warning of high-risk MGEs; informed stewardship	Early implementation/pilot projects	Medium–high	Requires standardized metadata, bioinformatic pipelines; cross-sector collaboration essential	[167,183]
Integrated One Health Stewardship	Multi-layered interventions	Combines clinical, veterinary, environmental measures to control ARGs/MGEs simultaneously	One Health: maximizes cross-sector impact; prevents ARG spillover	Conceptual/policy framework	Moderate–high if coordinated	Synergy of conventional and MGE-focused strategies; sustainable long-term approach; multi-sector integration needed	[166,181,194]

### 9.3. Antimicrobial Peptides (AMPs) to Prevent ARG Spread

Antimicrobial peptides (AMPs) represent a promising class of next-generation therapeutics for mitigating antimicrobial resistance (AMR) and limiting the dissemination of antibiotic resistance genes (ARGs) [195]. Unlike conventional antibiotics that typically target specific intracellular pathways, AMPs exert rapid bactericidal activity primarily through membrane disruption, pore formation, and multi-target intracellular interference [196]. This broad-spectrum and non-specific mode of action reduces the likelihood of resistance development and minimizes selective pressure that drives the mobilization of ARGs via mobile genetic elements (MGEs). Consequently, AMPs may indirectly suppress horizontal gene transfer (HGT) by limiting bacterial survival and reducing the ecological niches that favor MGE propagation [197].

AMPs are widely distributed across diverse biological systems, including animals, plants, and microorganisms, with marine biofilms emerging as particularly rich reservoirs of novel peptide candidates [196]. Their demonstrated efficacy against multidrug-resistant (MDR) pathogens, including carbapenem-resistant *Enterobacteriaceae* and *Acinetobacter baumannii*, underscores their therapeutic potential [198]. Compared to conventional antibiotics, AMPs exhibit faster killing kinetics and reduced resistance selection. While CRISPR-based systems and phage therapy offer high specificity in targeting ARGs or pathogens, AMPs provide a broader, less resistance-prone approach, making them attractive components of integrated strategies to control MGE-mediated resistance dissemination [196,198].

## 10. Recommendations

1. Strengthen One Health surveillance by integrating genomic tools across human, veterinary, and environmental sectors.
2. Develop MGE-targeted therapeutics, including conjugation inhibitors, integrase blockers, and CRISPR-based antimicrobials.
3. Limit selective pressures through prudent antimicrobial stewardship and reduction in co-selectors such as heavy metals and biocides.
4. Expand genomic databases for novel MGEs to enhance monitoring and risk assessment.
5. Promote international collaboration in data sharing, outbreak tracking, and containment of high-risk plasmids and resistance islands.

## 11. Conclusions

This review set out to examine the roles of mobile genetic elements in the acquisition, mobilization, and dissemination of antimicrobial resistance genes across clinical, environmental, and agricultural ecosystems. Our objective was to integrate molecular mechanisms with ecological and epidemiological insights, thereby elucidating how plasmids, integrons, transposons, ICEs, and phages collectively underpin the global AMR crisis. The evidence presented demonstrates that MGEs are not passive carriers but dynamic innovation hubs that drive recombination, assemble multidrug resistance clusters, and stabilize resistance in bacterial populations. These processes occur under strong selection pressures, including the misuse of antibiotics, the co-exposure to heavy metals, and global trade and travel. By framing MGEs within a One Health context, we emphasize their capacity to bridge human, animal, and environmental reservoirs, accelerating the spread of multidrug resistance beyond traditional clinical settings. Targeting MGEs is both necessary and promising. They drive the persistence and spread of AMR, while new tools such as metagenomics, resistome mobilome mapping, CRISPR, and phage therapy provide practical solutions for mitigation.

## Figures and Tables

**Table 2:** Representative conjugative systems, their genetic determinants, and associated ARGs.

Conjugative System	Genetic Determinants	Associated ARGs	References
IncF plasmids	*tra* operon (*traI*, *traD*, *traM*, *trb*)	*tetA*, *aac(3)-IIa*, *bla*_CTX-M_	[44]
IncI1 plasmids	pil operon (*thin pili*), shufflon region for pilus variety	*tetB*, *bla*_CMY-2_, *qnrS*	[45,46]
IncA/C2 plasmids	*tra* genes, *mob* genes large multidrug resistance regions	*sul1*, *aadA*, *tetA*, *bla*_NDM-1_	[47,48]
IncX3 plasmids	conserved transfer genes (*pilX*, *taxA*)	*bla*_OXA-181_, *bla*_NDM_,	[44,49]
IncHI2 plasmids	*tra* genes (transfer region 1 and 2), replication determinants	*bla*_CTX-M_, *floR*, *mcr-1*,	[48,50]
ICEs (Integrative and Conjugative Elements, e.g., SXT/R391 family)	*int* (integrase), *setR* (repressor), conjugation modules	*strA/B*, *sul2*, *dfrA*, *floR*,	[51,52]
Tn916/Tn1545 family (Conjugative Transposons)	*int*, *xis*, *orf20* (conjugation genes)	*aphA ermB*, *tetM*	[53,54]

**Table 3:** Mosaic MGEs and their associated ARG profiles.

Mosaic/Hybrid MGE	Genetic Features	Associated ARGs	References
Mosaic F-type plasmids	Backbones that are highly recombined with integrons and transposons.	*bla*_CTX-M_, *sul1*, *tetA*, *aac(6′)-Ib*	[8,68]
Hybrid plasmids in Gram-negative pathogens	Integration of prophage elements, integrons, and transposons.	*aadA, qnr, bla*_TEM_ *, bla*_SHV_	[14,69]
Mosaic ICEs (Integrative and Conjugative Elements)	Mobility modules, resistance cassettes, and integrases recombining	*dfrA*, *floR*, *sul2*	[26,70]
Tn3-family derived hybrid elements	Transposons coupled with plasmid modules and integrons.	*aac(3)-IIa*, *bla*_KPC_, *bla*_NDM_	[8,71]
Mosaic MGEs in Enterobacteriaceae	Plasmid recombination was used to incorporate ARGs.	*oqxAB*, *mcr-1*, *bla*_NDM_	[72]
MOB F12 A plasmids (F-type plasmid derivatives)	Mobile resistance modules on mosaic plasmid backbones	*tet genes*, *qnrS*, *bla*_CTX-M_	[69,73]
Hybrid SXT elements in Vibrio cholerae	SXT ICEs with components obtained from phages and plasmids	*strA*/*B*, *dfrA*, *sul2*	[74]

**Table 4:** Globally disseminated MGE-associated resistance genes: first reports, host bacteria, and health implications.

Resistance Gene	Associated MGEs	First Reported (Year, Location)	Host Bacteria (Examples)	Geographic Spread	Clinical/Public Health Significance	Reference
*bla*_NDM-1_	*IncA*/*C*, *IncFII*, *IncX3*, *IncHI1* plasmids	2008, India	*K. pneumoniae*, *E. coli*, *Enterobacter*	Global (Asia, Europe, Americas, Africa)	Contributes to carbapenem resistance; widespread among hospital and environmental isolates; high potential for horizontal transfer	[125,126]
*mcr-1*	*IncI2*, *IncX4*, *IncHI2* plasmids	2015, China (pigs)	*E. coli*, *Salmonella*, *Klebsiella*	Global (Asia, Europe, Americas, Africa)	First transferable colistin resistance; concerns for last-resort antibiotic efficacy; rapidly spread in food animals, humans, and environment	[127,128]
*tet(X3*/*X4)*	*IncQ1*, *IncFII*, *IncX1* plasmids; IS CR2	2019, China (animals)	*E. coli*, *Acinetobacter*	China, expanding globally	High-level tigecycline resistance; frequently co-occurs with carbapenemase genes; major concern in hospital settings	[129,130]
*bla*_KPC (Tn4401)_	Tn4401 on *IncFII*, *IncN*, *IncL* plasmids	2001, USA	*K. pneumoniae* (ST258), *Enterobacter*, *Citrobacter*	Global (USA, Europe, Latin America, Asia)	Major contributor to carbapenem resistance in hospital-acquired infections; widespread via horizontal transfer	[131,132]
*bla*_OXA-48_	Tn1999 on IncL/M plasmids	2001, Turkey	*K. pneumoniae*, *E. coli*	Middle East, Europe, North Africa, Asia	Silent dissemination; resistant to carbapenems; contributes to hospital outbreaks	[126,133]
*vanA*/*vanB*	Tn1546 (transposon) on plasmids	1986, Europe	*Enterococcus faecium*, *E. faecalis*	Global (hospitals, food chain, animals)	Vancomycin resistance in enterococci; zoonotic transfer; major concern for hospital infections	[134]
*qnr* genes	Plasmids with ISCR1 elements	Early 2000s, USA and Asia	*Enterobacteriaceae* (*E. coli*)	Global	Plasmid-mediated quinolone resistance; contributes to fluoroquinolone resistance	[135,136]
*bla*_CTX-M_ *ESBLs*	ISEcp1, IS26 transposition units; IncF plasmids	1990, Germany (CTX-M-1); Japan (CTX-M-2)	*E. coli*, *Klebsiella*, *Salmonella*	Worldwide, esp. community-acquired infections	Dominant ESBLs in hospitals and community; foodborne dissemination	[126,137]

**Table 5:** Detection, monitoring, and genomic surveillance methods for MGEs.

Method	Type of Detection	Principle	Applications	Pros	Cons/Limitations	Representative Tools/Resources	Typical Time Required	Key References
Conjugation Assays	Phenotypic/Functional	Mating donor and recipient strains on a filter or broth; select trans conjugants; calculate transfer frequency.	Determine plasmid/ICE mobility and host-range.	Functional, quantitative, detects co-transfer events.	Limited to culturable organisms; requires selectable markers.	Filter- and broth-mating protocols Standard recipients: *E. coli* J53, HB101	2–3 days (overnight mating + plating + colony counting)	[152]
PCR-based Replicon Typing (PBRT)	Molecular (PCR-based)	Multiplex PCR Detection of plasmid replicons using specific primers (amplification of replicon-specific DNA regions)	Plasmid typing, AMR surveillance, epidemiology, clinical profiling	Simple, rapid, cost-effective.	Limited to known replicons.	Custom primer sets, standard PCR reagents, agarose gel	6–8 h (PCR + gel)	[153]
S1-PFGE + ddPCR	Molecular/Genotypic (ddPCR)	S1 nuclease linearizes plasmids; PFGE separates them; ddPCR quantifies plasmid replicons and AMR genes	Plasmid replicon typing; AMR gene detection; epidemiological tracking	High sensitivity; quantitative; simplified workflow	Labor-intensive; specialized PFGE and ddPCR equipment required; limited to known targets	CHEF-DR III PFGE System (Bio-Rad) QX200 ddPCR System (Bio-Rad) Custom primers/probes	2–3 days (S1-PFGE 1–2 days, ddPCR 1 day)	[154]
Plasmid Extraction and Profiling	Phenotypic/Basic Molecular	Plasmids were isolated via alkaline lysis (Zymogen UK protocol) and resolved by agarose gel electrophoresis. Bands were visualized under UV to confirm presence and estimate size.	Detect plasmid-mediated antibiotic resistance and Assess MGE contribution to resistance dissemination.	Simple, inexpensive.	May lose large/low-copy plasmids.	Qiagen Plasmid Midi/Maxi Kit NucleoBond Xtra Midi	4–6 h (including electrophoresis)	[155]
PCR, Southern blot, Sequencing	Molecular/DNA-based	PCR amplifies target genes; Southern blot hybridizes labeled probes to target DNA fragments to confirm presence of MGEs; Sequencing analyzes MGE diversity	Detect and monitor plasmids/integrons; assess diversity; study pesticide impact on MGEs	Specific, confirms PCR via Southern blot, diversity profiling via sequencing	PCR may miss unknown MGEs; Southern blot laborious; sequencing costly/time-consuming	Plasmid/integron-specific primers; DIG-labeled probes; Pyrosequencing platform	8–17 days	[156]
WGS + geNomad	Molecular/Computational	Analyze whole-genome/metagenome sequences to detect plasmids and viruses using alignment-free and gene-based models	Identify MGEs, plasmid/virus diversity, track horizontal gene transfer, AMR surveillance	Comprehensive, high accuracy, scalable, detects both plasmids and viruses	Requires high-quality WGS data, computational resources, dependent on database completeness	geNomad, IMG/M database, sequencing platformsResFinder, CARD	Hours–days (depends on dataset size and compute power)	[157]
PlasmidFinder	Bioinformatics	Detects plasmid-specific replicon sequences in WGS data by alignment to a curated plasmid database	Rapid plasmid typing, AMR surveillance, epidemiology, tracking plasmid-mediated resistance	User-friendly, web and command-line options, fast, accurate for known plasmid types	Limited to known plasmid types; cannot reconstruct full plasmids	PlasmidFinder, pMLST	Minutes to a few hours depending on data size	[158]
ISfinder	Bioinformatics	Detects known IS elements by sequence homology; provides annotation and classification into families	IS identification, ARG mobilization studies, genome annotation	Curated database, standardized nomenclature, supports phylogeny and annotation	- Detects only known IS elements - Cannot predict activity or mobility - Novel IS elements require submission for inclusion	ISfinder	Minutes to hours (depends on genome size and analysis pipeline)	[159]
IntegronFinder	Bioinformatics	Detects integrons by identifying integrase genes and attC sites; finds gene cassettes	Mapping integrons, resistome analysis, and ARG mobilization studies	Accurate detection of characterized integrons supports genome-scale analysis	Limited to known/characterized integrons; may miss highly divergent cassettes	IntegronFinder	Minutes to hours (depends on genome size)	[160]
ICEberg	Bioinformatics database for ICEs/IMEs	Detects integrative and conjugative elements by identifying integrases and conjugation modules	Tracking conjugative chromosomal elements, studying ARG dissemination	Curated database; supports ICE/IME annotation and comparative genomics	Requires sequence context; may miss novel ICEs	ICEberg	Minutes to hours (depends on genome size)	[161]
Illumina (Short-read WGS)	Sequencing/Genomic	High-accuracy, paired-end short reads assembled into contigs; plasmid detection via marker search, assembly graph analysis, or contig classification	Detect plasmids; resistome profiling; SNP and small variant analysis; surveillance of ARGs	High base accuracy; cost-effective; high throughput; suitable for small plasmids and known plasmid types	Poor reconstruction of large/repetitive plasmids; fragmented assemblies; ARGs may not be assigned to plasmids; novel plasmids can be missed	PlasmidSPAdes, Recycler, cBar, PlasmidFinder	Library prep and sequencing: 1–2 days; Analysis: hours–1 day per genome	[162]
Oxford Nanopore Technologies (ONT) Long-Read Sequencing	Genomic/Assembly-based	Sequencing of single DNA molecules through nanopores generates long reads (>10 kb, often >50 kb) spanning entire MGEs and their flanking sites, allowing direct assembly and structural resolution.	- Detection and reconstruction of full-length MGEs (ICEHo-I/II, IS elements, transposons, prophages). - Determination of integration sites and genome context	- Long reads resolve repetitive MGE regions that short reads cannot. - Portable and scalable platform (MinION/GridION). - Real-time data generation allows early analysis.	- Higher raw error rate (~5–10%) than Illumina (needs polishing for SNP-level accuracy). - Requires high molecular weight DNA for ultra-long reads. - Sequencing yield may vary depending on flow cell quality.	ONT platform (MinION/GridION), Basecalling (Guppy), Assemblers (Canu, Flye), Visualization (Mauve)	Library prep + sequencing run: ~24–48 h (depending on coverage); Assembly: few hours	[163]
PacBio SMRT sequencing (HiFi reads)	Sequencing-based (long-read)	Single-molecule real-time sequencing generates very long reads (>10–20 kb, HiFi can reach >99% accuracy) that span repetitive regions and full plasmids, allowing direct detection and assembly of MGEs (plasmids, prophages, genomic islands, secretion system modules).	Detection and complete assembly of plasmids and chromosomal MGEs; identification of prophage regions, integrative elements, secretion systems, resistance operons.	Produces highly contiguous assemblies; resolves repeats and structural variants; allows closed plasmid sequences; ideal for MGEs with repeats or nested elements; HiFi improves base accuracy.	Requires high-quality DNA; more expensive than short reads; lower throughput; some smaller variants may need additional polishing or hybrid Illumina reads for error correction.	PacBio Sequel II/SMRT platform; HGAP/SMRT Link pipelines for assembly; tools for downstream MGE annotation: PGAP, PHASTER, RAST.	Library prep and sequencing: 1–3 days; assembly/polishing: hours–1 day.	
Hybrid Assembly (Short + Long Reads)	Genomic/NGS	Combine Illumina + ONT/PacBio reads for complete assemblies.	Accurate reconstruction of plasmids/ICEs.	Resolves repeats and structural variants.	Costlier; bioinformatics expertise required.	Unicycler, Flye, Pilon, Trycycler	1–2 weeks (DNA prep + two sequencing runs + assembly/polishing)	[164]
Plasmidomics/Metagenomic Approaches	Metagenomic/Culture-independent	Assembly of metagenomic reads to reconstruct plasmid-like sequences (PLSs) and identify MGEs based on replicons, integrases, and sequence motifs	Detect MGEs from complex microbiomes.	Captures non-culturable plasmids.	DNA bias, computationally demanding.	MOB-suite, PlasFlow, PlaScope, MetaPlasm	2–3 weeks (data acquisition + assembly + analysis)	[165]

## Data Availability

No new data were created or analyzed in this study. Data sharing is not applicable to this article.

## Abbreviations

The following abbreviations are used in this manuscript:

AMR	Antimicrobial Resistance
ARGs	Antibiotic Resistance Genes
MGEs	Mobile Genetic Elements
HGT	Horizontal Gene Transfer
MDR	Multidrug-Resistant
XDR	Extensively Drug-Resistant
ICEs	Integrative and Conjugative Elements
IMEs	Integrative and Mobilizable Elements
ISs	Insertion Sequences
ISCR	Insertion Sequence Common Region
MITEs	Miniature Inverted-repeat Transposable Elements
PICIs	Phage-Inducible Chromosomal Islands
CRISPR	Clustered Regularly Interspaced Short Palindromic Repeats
dsDNA	Double-stranded DNA
ESBL	Extended-Spectrum Beta-Lactamase
MICs	Middle-Income Countries
LICs	Low-Income Countries
PBPs	Penicillin-Binding Proteins
SOS	Save Our Souls (bacterial global DNA damage response system)
WWTPs	Wastewater Treatment Plants
TA	Toxin–Antitoxin
BHR	Broad-Host-Range
T4SS	Type IV Secretion System
oriT	Origin of Transfer
*rep*	Replication Gene
*tra*	Transfer Gene
*mob*	Mobilization Gene
int	Integrase
xis	Excisionase
Pc	Promoter for Cassette Expression in Integrons
